# Association between mental demands at work and cognitive functioning in the general population – results of the health study of the Leipzig research center for civilization diseases (LIFE)

**DOI:** 10.1186/1745-6673-9-23

**Published:** 2014-05-28

**Authors:** Francisca S Then, Tobias Luck, Melanie Luppa, Katrin Arélin, Matthias L Schroeter, Christoph Engel, Markus Löffler, Joachim Thiery, Arno Villringer, Steffi G Riedel-Heller

**Affiliations:** 1 Institute of Social Medicine, Occupational Health and Public Health (ISAP), Medical Faculty, University of Leipzig, Philipp-Rosenthal-Str. 55, 04103 Leipzig, Germany; 2 LIFE – Leipzig Research Center for Civilization Diseases, University of Leipzig, Leipzig, Germany; 3 Max-Planck-Institute for Human Cognitive and Brain Sciences, Leipzig, and Clinic for Cognitive Neurology, University Hospital Leipzig, Leipzig, Germany; 4 Institute for Medical Informatics, Statistics and Epidemiology (IMISE), University of Leipzig, Leipzig, Germany; 5 Institute of Laboratory Medicine, Clinical Chemistry and Molecular Diagnostics (ILM), University of Leipzig, Leipzig, Germany

**Keywords:** Cognition, Cognitive abilities, Occupation, Work, Demands, Self-regulation, Population-based study

## Abstract

**Background:**

The level of mental demands in the workplace is rising. The present study investigated whether and how mental demands at work are associated with cognitive functioning in the general population.

**Methods:**

The analysis is based on data of the Health Study of the Leipzig Research Centre for Civilization Disease (LIFE). 2,725 participants aged 40–80 years underwent cognitive testing (Trail-Making Test, Verbal Fluency Test) and provided information on their occupational situation. Participants over the age of 65 years additionally completed the Mini-Mental State Examination. Mental demands at work were rated by a standardized classification system (O*NET). The association between mental demands and cognitive functioning was analyzed using Generalized Linear Modeling (GENLIN) adjusted for age, gender, self-regulation, working hour status, education, and health-related factors.

**Results:**

Univariate as well as multivariate analyses demonstrated significant and highly consistent effects of higher mental demands on better performance in cognitive testing. The results also indicated that the effects are independent of education and intelligence. Moreover, analyses of retired individuals implied a significant association between high mental demands at work of the job they once held and a better cognitive functioning in old age.

**Conclusions:**

In sum, our findings suggest a significant association between high mental demands at work and better cognitive functioning. In this sense, higher levels of mental demands – as brought about by technological changes in the working environment – may also have beneficial effects for the society as they could increase cognitive capacity levels and might even delay cognitive decline in old age.

## Introduction

There is a continuous trend of rising mental demands in the general working environment, particularly in economically advanced countries [1]. The shift away from physical jobs to occupations with higher mental demands apparently encompasses all educational groups [2]. The motor of this shift seems to be skill-based technological changes which bring about a greater contingent of professional, technical, and associate jobs [1]. The ongoing accelerated technological advances push companies to compete at increased quality standards which then involve higher mental demands for the worker [3]. The trend of increasing levels of mental demands in the working environment raises the question of how mental work demands may affect the individual.

High mental demands encountered on a daily level, as in the workplace, might exert a harmful or beneficial effect on cognitive functioning. Experimental studies, for example, have shown that very demanding tasks lead to decrements of performance in cognitive testing (e.g., [4-6]). Moreover, high mental demands might embody a stressor that, if chronic, could impair cognitive functioning [7]. However, studies have shown that whether high mental demands really impair cognitive performance depends on task difficulty and neuroendocrinological effects [8]. Hence, it is possible that – under particular conditions – high mental demands can affect cognitive functioning in a harmful manner. On the other hand, there is evidence that high mental demands may have a beneficial effect on cognitive functioning. Findings of epidemiologic studies in the elderly (aged 70 years or older) imply that individuals with mentally demanding jobs may have a lower risk of cognitive impairment in old age [9,10] and a reduced risk of dementia [11,12]. Similarly, studies examining manual work [13,14] or complexity of work tasks [15,16] suggest that lower mental demands at work may be associated with a higher dementia risk. Essentially, mental activity throughout the life-course is thought to build up a cognitive reserve that enhances the capacity of the cerebral neural network to efficiently compensate for pathogenic damages and, thus, delays the symptomatic onset of severe neuropsychiatric diseases such as dementia [17]. The motivational reserve model adds another component to the cognitive reserve: It argues that innate motivation determines the amount of highly demanding conditions confronted with during life [18]. Hence, a high motivational reserve seems to reduce the risk of cognitive impairment in old age [19]. In light of the available research results, we are faced with two sets of studies predicting either a harmful or a beneficial effect of mental demands at work on cognitive functioning.

Considering evidence on a beneficial effect of high mental demands on cognitive functioning, we need to be aware that these studies have all been conducted in the elderly population. Evidence for younger individuals is extremely sparse – nonetheless, it is extremely important because, if mental demands impact cognitive functioning, then they do that already when the individuals are still an active part of the workforce. Moreover, evidence on a beneficial effect of high mental demands on cognitive functioning in population-based studies commonly involves threshold level examinations (either by a clinical diagnosis or by using cutoffs). These types of analyses may be difficult to interpret in particular for individuals with initially poorer performance skills (e.g., low education). Analyzing effects on cognitive functioning in a dimensional space (using metric variables; as experimental studies do) could add valuable insights. Hence, the present study aimed at examining cognitive functioning via dimensional measures in a population-based sample of individuals aged 40–80 years old.

In addition, we considered it as relevant that the present study applied a comprehensive and objective definition of mental demands at work. Previous studies investigated either very specific types of mental demands at work (i.e., complexity with people, time pressure, etc.) or a crude indicator of mental demands (i.e., manual vs. non-manual labor, Dictionary of Occupational Titles, etc.). Then again other studies used subjective assessment methods for job demands by asking the participants, for example, how intellectually stimulating they perceive their job to be. Subjective assessment of job demands, however, could be biased. In the present study, we therefore focused on a comprehensive as well as objective indication of mental demands at work.

As the currently available evidence on high mental demands predicts two different outcomes, either a detrimental or a beneficial effect on cognitive functioning, the present study investigated the association between mental demands at work and cognitive functioning in a representative sample of the general population aged 40–80 years old.

## Methods

### Study design

The health study of the Leipzig Research Centre for Civilization Diseases (LIFE) is a large population-based study of a representative sample of the inhabitants of the city of Leipzig in Germany. An age- and gender-stratified random selection of inhabitants aged 40 to 80 years old was obtained from the population registry office. The target sample of the LIFE study is 10,000 participants with 1,250 participants per 5-year age group of which 50% are male and 50% are female. The only exclusion criterion is not being pregnant. Letters of invitation to participate in the study were sent out by mail. By February 2013, 11,825 letters of invitation were sent out. Of the invited individuals, 3,101 already participated in the study and about 30% refused participation. The recruitment process is still ongoing and response of the remaining invited individuals is pending. At the study center, the participants filled out an informed consent form. They then underwent a set of assessment batteries, including structured interviews (socio-demographic information, medical history, medications, and lifestyle factors like tobacco, alcohol, physical activity, sleep, eating behavior) and medical examinations (anthropometry, blood samples, cognitive functioning, heart anatomy & functioning, allergy, eye assessment, and others). The participants received a financial compensation of 20€.

The study was approved by the ethics committee of the University of Leipzig.

### Assessment of cognitive functioning

The assessment of cognitive functioning was always performed in the morning in an interview setting in a separate enclosed room. The instructions for the examiners were computerized and the examiners documented the participants’ results in an electronic data mask. All participants at the LIFE study center were asked to take part in the interview assessing cognitive performance level via the Trail Making Test (TMT) and the Verbal Fluency Test (VFT) - both subtests of the neuropsychological test battery of the Consortium to Establish a Registry for Alzheimer’s disease (CERADplus) [20-22]. All participants aged 65 years or older were asked to take part in an additional cognitive assessment at another day, which included the Mini-Mental State Examination (MMSE). The TMT is a test measuring the cognitive abilities working memory, task-switching ability [23,24] and executive control [25,26]. Furthermore, the TMT is an extensively used neuropsychological assessment for individuals of all age groups [24,27]. In the TMT, the participants have to connect numbers in an ascending order as fast as they can. The version B of the TMT has an increased task difficulty: The participants have to connect numbers and letters alternatingly. The participant’s score is the number of seconds needed to complete the test. A smaller score signifies a better cognitive functioning and a high score a poor cognitive functioning. The VFT measures verbal abilities, semantic fluency, and semantic memory [28,29]. In clinical practice, the VFT is used to assess cerebral lesions and progressive degenerating disorders for patients of all ages [30,31]. In the VFT, the participants have to name as many animals as possible in one minute. The score equals the number of correct animals named. A higher score represents a better cognitive functioning and a low score indicates a poor cognitive functioning. The MMSE is a screening tool for dementia and was completed only by participants over the age of 65 years. It contains 30 items that assess the level of global cognitive functioning. The highest possible score is 30, which indicates better cognitive functioning.

Participants who had difficulties completing the test (visual impairments, tremor, exhausted from a nightshift, etc.) or who had difficulties understanding the instructions due to language problems or with interruptions during the testing session were excluded from the analysis (n = 91). We also excluded individuals with chronic neuropsychiatric diseases/disorders, i.e., dementia, Parkinson’s disease, epilepsy, polyneuropathy, muscle atrophy, multiple sclerosis, schizophrenia, narcolepsy, or others (n = 83).

### Occupational information

Information on the participants’ occupation was obtained in personal standardized interview. The participants were asked “Are you temporarily working?”, “If yes, how many hours a week are you working?”, and “What occupational title has the job that you are/were mainly working in?”.

For the analysis, we first matched the participants' occupations to O*NET 17.0 standard occupational classification categories (http://www.onetonline.org). The O*NET database was developed by the US Department of Labor/Employment and Training Administration (USDOL/ETA) and provides a comprehensive set of occupational descriptors indicating worker characteristics, worker requirements, experience requirements, occupational requirements, workforce characteristics, and occupation-specific information for every type of occupation. The process of matching the participants’ occupations to O*NET codes was subject to stringent criteria such as corresponding task descriptions, comparable levels of responsibilities, and equivalent technical equipment used. Some participants could not be matched to an O*NET code since they did not provide their occupational title (n = 32) or there was no corresponding occupational group in the O*NET database (n = 10). These occupational groups included “master”, “helping my husband”, “service provider”, “demonstration worker”, and “expert”. Furthermore, some O*NET codes (e.g., 11–9199.00 “Managers, All Other”, 21–2011.00 “Clergy”, 15–1143.01 “Telecommunications Engineering Specialists”) had an incomplete set of O*NET descriptors and hence could not be used for analysis. All together n = 202 participants had to be excluded from analysis due to incomplete occupational data.

In a second step, we created an index of mental demands by using all O*NET descriptor variables of “Cognitive Abilities” at work (O*NET variables 1.A.1.a - 1.A.1.g.2, see Appendix). In order to obtain the index of mental demands, the average of these variables was calculated. The cronbach’s alpha of the index of mental demands was 0.97, respectively. We then categorized the mental demands in three categories: high, medium, and low demands.

In a last step, we additionally created an index of self-regulation at work by calculating the averages of the O*NET descriptor variables that describe job requirements with a high degree of self-regulation (O*NET variables 1.C.4.a Self-Control, 1.C.4.b Stress-Tolerance, 1.C.4.c Adaptability/Flexibility). The cronbach’s alpha of the self-regulation index was 0.88. We also categorized the self-regulation index in three categories: high, medium, and low.

### Statistical analyses

All statistical analyses were conducted using SPSS (Version 20).

To test for group differences, Mann–Whitney-U-Tests, Kruskal-Wallis-Tests, and Pearson’s x^2^-tests were performed as appropriate. The intercorrelation of the dependent variables was significant but the coefficients were low (see Table 1). Yet in order to avoid problems of multicollinearity, we (i) categorized the dependent variables, (ii) used a very large sample size, and additionally (iii) combined the variables demands and education in one variable.

**Table 1:** Correlations between the dependent variables

	**Mental demands**	**Age**	**Self-regulation**	**Intelligence**	**Working hours status**	**Education**
**Mental demands**^**§**^	1	0.169***	0.183***	0.280***	0.056**	0.461***
**Age**^**§**^	0.169***	1	−0.071***	0.178***	0.719***	0.092***
**Self-regulation**^**§**^	0.183***	−0.071***	1	0.084***	−0.086***	0.226***
**Intelligence**^**§**^	0.280***	0.178***	0.084***	1	0.035	0.452***
**Working hour status**^**#**^	0.056***	0.719***	−0.086***	0.035	1	−0.032
**Education**^**#**^	0.461***	0.092***	0.226***	0.452***	−0.032	1

*Notes:*^§^ - Pearson’s correlation, ^#^ - Spearman-Rho correlation, level of significance: **p < 0.01, ***p < 0.001.

Associations between mental demands and cognitive functioning were analyzed using Generalized Linear Modeling (GENLIN). An advantage of GENLIN is that it can be performed even when the distribution deviates from normal. In Model 1, the main effect of mental demands (low/medium/high) on cognitive functioning (score in TMT-B and VFT) was investigated. In Model 2, main effects of mental demands (low/medium/high) were adjusted for age (40-49/50-59/60-69/70-79), gender (female/male), self-regulation (low/medium/high), working hours status (not working/<15 h per week/15-34 h per week/35 + h per week), the level of education completed (primary/secondary/tertiary), having had a stroke (yes/no), having had a heart attack (yes/no), having had a liver disease (yes/no), and having high blood pressure (yes/no), elevated blood lipids (yes/no), and diabetes (yes/no). Information on the individuals’ health was obtained in a personal interview by the questions “Have you even been diagnosed with stroke/ heart attack/ liver disease/ high blood pressure/ elevated blood lipids/ diabetes?” In Model 3, we additionally analyzed additive effects of the level of education and mental demands. An additive score of education and demands was created by 1) scoring education and mental demands and 2) adding the scores. Scores were the following: primary education = 1, secondary education = 2, tertiary education = 3, low demands = 1, medium demands = 2, high demands = 3. The scores were added, so that each individual received one additive score for education and demands. For example, an individual with only primary education (=1) and a job with low mental demands (=1) received an additive score of 2. A person with completed tertiary education (=3) and a job with high mental demands (=3) received an additive score of 6. Possible additive scores comprised: 2 – primary education and low demands, 3 – primary education and medium demands, or secondary education and low demands, 4 – primary education and high demands, or secondary education and medium demands, or tertiary education and low demands, 5 – secondary education and high demands, or tertiary education and medium demands, 6 – tertiary education and high demands.

We conducted two further analyses: First, we addressed the potential impact of intelligence on the association between mental demands and cognitive functioning. Thus, we additionally adjusted Model 3 for estimated intelligence as measured by the German Version of the Mill Hill Vocabulary Scale [32]. Secondly, we were interested in possible long-term effects of mental demands at work on cognitive functioning. In order to evaluate whether there might be a long-term effect, we included only participants that were over the age of 65 and examined the association between the level of mental demands of the job that they had before retirement and current cognitive functioning as measured by the MMSE. The association was analyzed in the same types of models (Models 2 &-3) as described above.

## Results

Within the final sample (n = 2,725), 13.9% of the participants had jobs with high mental demands, 84.3% jobs with medium mental demands, and 12.6% jobs with low mental demands. Examples of jobs with high mental demands included chief executives, physicists, civil engineers, manufacturing engineering technologists, mathematicians, and others. Medium levels of mental demands at work are faced by participants in jobs such as administrative service managers, archivists, graphic designers, dispatchers, preschool teachers, or electrical power-line installers, etc. Jobs with low mental demands were, for example, stock clerks, packers, slaughterers, janitors, bartenders, or maids.

Within the group of participants in jobs with high mental demands, 9.6% had completed primary education, 24.6% secondary education, and 65.8% tertiary education. Of participants in jobs with medium levels of mental demands, 36.2% had completed primary education, 30.9% secondary education, and 32.8% tertiary education. Regarding, however, the participants in jobs with low mental demands, 66.9% had completed primary education, 27.9% secondary education, and 5.2% tertiary education.

The prevalence of having had a stroke, a heart attack, liver disease, high blood pressure or elevated blood lipids did not differ significantly by the level of mental demands at work (see Table 2). However, individuals in jobs with medium levels of mental demands had significantly lower rates of diabetes (10.8%) than individuals in jobs with low (14.9%) or high metal demands (15.3%).

**Table 2:** Prevalence of health-related events by the level of mental demands at work

	**Diabetes**	**High blood pressure**	**Stroke**	**Liver disease**	**Elevated blood lipids**	**Stroke**
Mental demands (low)	14.9%**	48.0%	4.3%	8.6%	40.6%	3.4%
Mental demands (middle)	*10.8%*	48.9%	2.7%	11.8%	36.2%	2.1%
Mental demands (high)	*15.3%*	52.1%	3.4%	12.3%	40.0%	2.4%
Demands & education (low & low)	14.9%*	48.5%*	5.1%	9.0%	44.0%	3.8%
Demands & education (middle & low)	*13.0%*	*49.2%*	2.5%	9.6%	37.8%	2.7%
Demands & education (middle & middle OR high & low)	*11.2%*	*49.9%*	3.3%	12.6%	35.6%	1.5%
Demands & education (high & middle)	*9.0%*	*47.3%*	2.3%	12.4%	34.3%	2.2%
Demands & education (high & high)	*16.1%*	*54.4%*	4.0%	14.0%	42.4%	2.0%

*Notes*: level of significance by Pearson’s x^2^-test: *p < 0.05, **p < 0.01, ***p < 0.001.

**Figure 1:**
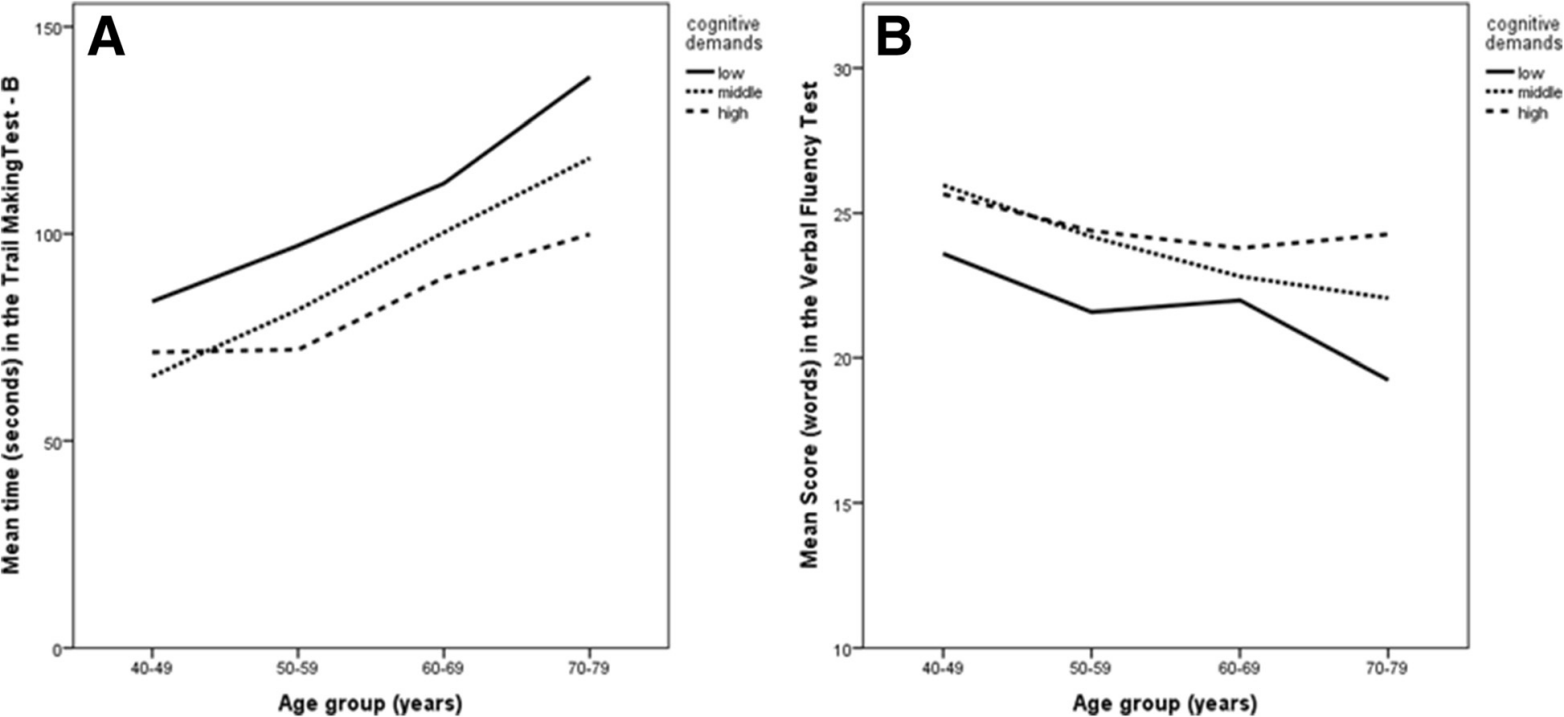
**Means in cognitive testing (Trail Making Test - B and Verbal Fluency Test) by the level of demands over the age groups. A**. Mean seconds needed to complete the Trial Making Test - B with lower scores indicating a better performance. **B**. Mean number of words named in the Verbal Fluency Test with higher scores indicating a better performance.

Findings on cognitive functioning of the study population are shown in Table 3. Participants who worked in jobs with high mental demands had significantly better scores in the cognitive tests TMT-B and VFT than participants who worked in jobs with low mental demands (p < 0.001). Moreover, participants who were younger, female, who worked fulltime, had a better health status, a higher education, and worked in a job with a higher self-regulation level performed also significantly better in the cognitive tests (p < 0.01) (Figure 1).

**Table 3:** Cognitive functioning of the study sample according to socio-demographic and work-related characteristics

	** n (%)**	**Trail making test - B mean (SD)**	**Verbal fluency mean (SD)**
Female^§^	1500 (51.2)	87.98 (45.64)***	23.85 (6.12)**
Male	1427 (48.8)	*97.97 (52.70)*	*23.20 (6.32)*
Age (70–79 years)^#^	680 (23.2)	116.79 (57.00)***	22.19 (5.86)***
Age (60–69 years)	867 (29.6)	*99.81 (49.00)*	*22.92 (5.95)*
Age (50–59 years)	776 (26.5)	*82.96 (40.40)*	*23.74 (6.30)*
Age (40–49 years)	604 (20.6)	*68.58 (35.09)*	*25.64 (6.31)*
Education (high)^#^	957 (32.9)	82.33 (38.06)***	25.24 (6.25)***
Education (middle)	836 (28.7)	*93.40 (46.71)*	*23.61 (5.92)*
Education (low)	1119 (38.2)	*101.66 (57.87)*	*22.04 (6.06)*
Not working^#^	1487 (51.1)	109.22 (55.20)***	22.41 (5.95)***
Working <15h/week	54 (1.8)	*95.20 (53.67)*	*23.84 (6.00)*
Working 15-34h/week	275 (9.4)	*78.67 (36.64)*	*25.44 (6.83)*
Working 35 + h/week	1096 (37.6)	*74.30 (34.00)*	*24.61 (6.17)*
Demands (high)^#^	379 (13.9)	87.58 (38.85)***	24.31 (6.34)***
Demands (medium)	1997 (84.3)	*91.59 (48.85)*	*23.70 (6.13)*
Demands (low)	344 (12.6)	*105.52 (59.78)*	*21.69 (6.06)*
Self-regulation (high)^#^	199 (7.3)	82.09 (41.54)***	26.04 (6.09)***
Self-regulation (medium)	2295 (84.3)	*92.18 (48.63)*	*23.48 (6.17)*
Self-regulation (low)	230 (8.4)	*108.08 (58.75)*	*21.91 (5.99)*
Stroke (no)^§^	2819 (97.7)	92.10 (48.18)***	23.61 (6.19)***
Stroke (yes)	66 (2.3)	*130.27 (81.01)*	*21.14 (7.10)*
Blood lipids (normal)^§^	1665 (63.0)	89.88 (48.23)***	23.98 (6.23)***
Blood lipids (elevated)	976 (37.0)	*99.26 (51.52)*	*22.99 (6.17)*
Liver disease (no)^§^	2368 (88.7)	93.09 (50.15)**	*23.64 (6.26)*
Liver disease (yes)	301 (11.3)	*98.32 (49.61)*	*23.09 (5.96)*
Heart attack (no)^§^	2792 (69.9)	92.36 (49.48)***	23.62 (6.20)*
Heart attack (yes)	88 (3.1)	*109.82 (45.44)*	*21.89 (6.39)*
Blood pressure (normal)^§^	1391 (50.9)	85.61 (45.16)***	24.16 (6.29)***
Blood pressure (high)	1340 (49.1)	*100.98 (52.55)*	*22.96 (6.09)*
Diabetes (no)^§^	2534 (88.0)	90.87 (48.37)***	23.75 (6.23)***
Diabetes (yes)	346 (12.0)	*108.01 (54.74)*	*22.13 (5.91)*

*Notes: SD* standard deviation, ^§^ - Mann–Whitney-U-Test, ^#^ - Kruskal-Wallis-Test, level of significance: *p < 0.05, **p < 0.01, ***p < 0.001.

Findings of univariate analyses using GENLIN modeling supported such a significant association between higher mental demands and better cognitive functioning (p < 0.001 for scores in the TMT-B and VFT; see Table 4, Model 1). The associations remained significant after adjustment for age, gender, self-regulation, working status, level of education, having had a stroke, heart attack, liver disease, high blood pressure, elevated blood lipids, and diabetes (see Table 4, Model 2). In the multivariate model (Model 2), also a high level of education and a younger age was also significantly associated with better scores in cognitive testing. Moreover, we found significantly lower scores in the TMT-B in individuals with male gender and who have had a stroke or were not working (see Table 4).

**Table 4:** Generalized linear model analysis on the effect of mental demands at work on cognitive functioning

	**Model 1**				**Model 2**					**Model 3**			
	**Trail making test - B**		**Verbal fluency**		**Trail making test - B**		**Verbal fluency**			**Trail making test - B**		**Verbal fluency**	
	**β**	**p**	**β**	**P**	**β**	**p**	**β**	**p**		**β**	**p**	**β**	**P**
Constant												4.659	0.000	3.077	0.000	4.437	0.000	3.126	0.000	Constant	4.422	0.000	3.130	0.000
Demands (high)												*−0.186*	*0.000*	*0.114*	*0.000*	*−0.161*	*0.000*	*0.068*	*0.002*	Demands & education (high & high)	*−0.346*	*0.000*	*0.192*	*0.000*
Demands (medium)												*−0.141*	*0.000*	*0.089*	*0.000*	*−0.096*	*0.000*	*0.047*	*0.005*	Demands & education (high & middle)	*−0.257*	*0.000*	*0.158*	*0.000*
Demands (low)												REF		REF		REF		REF		Demands & education (middle & middle OR high & low)	*−0.160*	*0.000*	*0.094*	*0.000*
Age (70–79 years)												*0.406*	*0.000*	*−0.120*	*0.000*	Demands & education (middle & low)	*−0.067*	*0.036*	*0.048*	*0.020*				
Age (60–69 years)												*0.292*	*0.000*	*−0.098*	*0.000*	Demands & education (low & low)	REF		REF					
Age (50–59 years)												*0.189*	*0.000*	*−0.076*	*0.000*	Age (70–79)	*0.406*	*0.000*	*−0.121*	*0.000*				
Age (40–49 years)												REF		REF		Age (60–69)	*0.291*	*0.000*	*−0.098*	*0.000*				
Female												*−0.103*	*0.000*	0.019	0.078	Age (50–59)	*0.188*	*0.000*	*−0.076*	*0.000*				
Male												REF		REF		Age (40–49)	REF		REF					
Self-regulation (high)												−0.071	0.089	*0.080*	*0.003*	Female	*−0.101*	*0.000*	0.019	0.078				
Self-regulation (medium)												−0.029	0.338	0.016	0.410	Male	REF		REF					
Self-regulation (low)												REF		REF		Self-regulation (high)	−0.079	0.058	*0.081*	*0.003*				
Not working												*0.167*	*0.000*	−0.028	0.090	Self-regulation (medium)	−0.033	0.269	0.014	0.478				
Working <15h/week												*0.145*	*0.023*	−0.020	0.624	Self-regulation (low)	REF		REF					
Working 15-34h/week												0.048	0.107	0.042	0.026	Not working	*0.169*	*0.000*	−0.029	0.085				
Working 35 + h/week												REF		REF		Working <15h/week	*0.144*	*0.024*	−0.020	0.610				
Education (high)												*−0.194*	*0.000*	*0.120*	*0.000*	Working 15-34h/week	0.050	0.093	*0.042*	*0.027*				
Education (medium)												*−0.070*	*0.001*	*0.052*	*0.000*	Working 35 + h/week	REF		REF					
Education (low)												REF		REF		Stroke (yes)	*0.115*	*0.038*	−0.028	0.414				
Stroke (yes)												*0.112*	*0.042*	−0.028	0.428	Stroke (no)	REF		REF					
Stroke (no)												REF		REF		Blood lipids (elevated)	−0.015	0.393	−0.006	0.568				
Blood lipids (elevated)												−0.015	0.397	−0.006	0.580	Blood lipids (normal)	REF		REF					
Blood lipids (normal)												REF		REF		Liver disease (yes)	0.022	0.379	−0.004	0.817				
Liver disease (yes)												0.022	0.380	−0.004	0.787	Liver disease (no)	REF		REF					
Liver disease (no)												REF		REF		Heart attack (yes)	0.009	0.842	−0.026	0.380				
Heart attack (yes)												0.006	0.890	−0.027	0.370	Heart attack (no)	REF		REF					
Heart attack (no)												REF		REF		Blood pressure (high)	0.009	0.617	0.000	0.970				
Blood pressure (high)												0.009	0.597	0.000	0.984	Blood pressure (normal)	REF		REF					
Blood pressure (normal)												REF		REF		Diabetes (yes)	0.019	0.474	−0.027	0.101				
Diabetes (yes)												0.018	0.500	−0.026	0.117	Diabetes (no)	REF		REF					
Diabetes (no)												REF		REF										

*Notes:* β - regression coefficient, p - level of significance, *REF* reference category.

**Figure 2:**
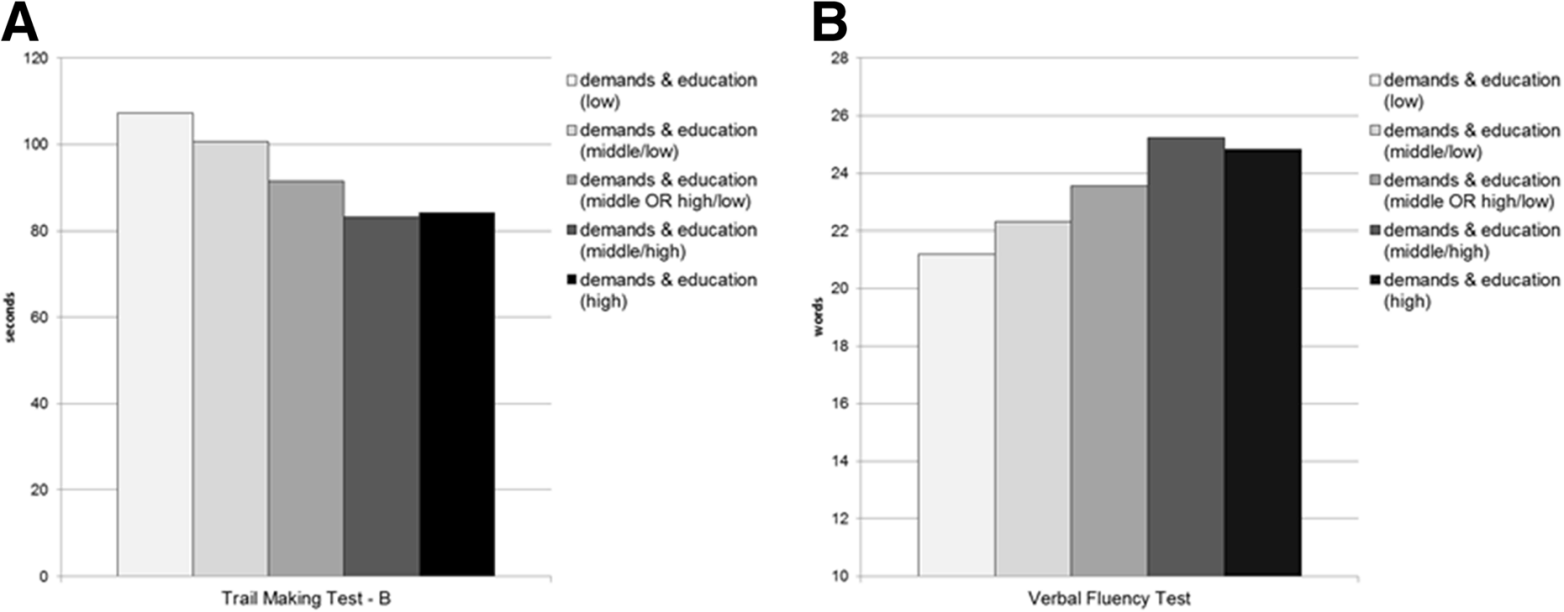
**Means in cognitive testing (Trail Making Test - B and Verbal Fluency Test) by the level of demands and education. A**. Mean seconds needed to complete the Trial Making Test - B with lower scores indicating a better performance. **B**. Mean number of words named in the Verbal Fluency Test with higher scores indicating a better performance.

In a third GENLIN model, we inspected a potential additive effect of mental demands and education on cognitive functioning (Model 3). We observed a highly significant additive effect of mental demands and education on better performance in the TMT-B and VFT (p < 0.001; see Table 4). The effect sizes of the factors in Model 3 were comparable to those in Model 2. The means in cognitive testing (TMT-B, VFT) by the level of demands and education are shown in Figure 2.

In order to dismiss a potential influence of intelligence on the association between mental demands and cognitive functioning, we included intelligence as independent variable in the GENLIN Model 3. The association between mental demands as well as education and cognitive functioning remained significant after including IQ in the model (see Table 5).

**Table 5:** Generalized linear model analysis on the effect of mental demands at work on cognitive functioning, including intelligence as covariate

	**Model 3**			
	**Trail making Test - B**		**Verbal fluency**	
	**β**	**p**	**β**	**P**
Constant	4.473	0.000	3.070	0.000
Demands & education (high & high)	*−0.189*	*0.000*	*0.135*	*0.000*
Demands & education (high & middle)	*−0.124*	*0.001*	*0.092*	*0.000*
Demands & education (middle & middle OR high & low)	*−0.072*	*0.052*	*0.062*	*0.018*
Demands & education (middle & low)	*−0.046*	*0.202*	*0.035*	*0.167*
Demands & education (low & low)	REF		REF	
Age (70–79)	*0.312*	*0.000*	*−0.113*	*0.000*
Age (60–69)	*0.286*	*0.000*	*−0.101*	*0.000*
Age (50–59)	*0.170*	*0.000*	*−0.063*	*0.000*
Age (40–49)	REF		REF	
Female	*−0.104*	*0.000*	0.030	0.015
Male	REF		REF	
Self-regulation (high)	−0.053	0.235	0.057	0.071
Self-regulation (medium)	−0.004	0.916	−0.005	0.833
Self-regulation (low)	REF		REF	
Not working	*0.076*	*0.003*	0.002	0.928
Working <15h/week	−0.086	0.235	−0.045	0.365
Working 15-34h/week	−0.031	0.277	*0.044*	*0.031*
Working 35+h/week	REF		REF	
Intelligence (high)	*−0.296*	*0.000*	*0.220*	*0.000*
Intelligence (middle)	*−0.187*	*0.000*	*0.119*	*0.000*
Intelligence (low)	REF		REF	
Stroke (yes)	−0.038	0.572	0.020	0.662
Stroke (no)	REF		REF	
Blood lipids (elevated)	0.008	0.671	−0.021	0.106
Blood lipids (normal)	REF		REF	
Liver disease (yes)	0.037	0.180	−0.001	0.974
Liver disease (no)	REF		REF	
Heart attack (yes)	−0.048	0.420	0.029	0.493
Heart attack (no)	REF		REF	
Blood pressure (high)	0.012	0.541	−0.003	0.850
Blood pressure (normal)	REF		REF	
Diabetes (yes)	0.033	0.272	−0.038	0.076
Diabetes (no)	REF		REF	

*Notes:* β - regression coefficient, p - level of significance, REF - reference category.

In a last step, we were interested in possible long-term effects of mental demands at work on cognitive functioning. Thus, in a subset of participants that were over the age of 65 years (mean age = 71; n = 422), we examined the association between the level of mental demands of the job that they had before retirement and current cognitive functioning as measured by the MMSE. Results are shown in Table 6 (Model 4 and 5). Model 4 shows significant associations between higher MMSE scores and high mental demands at work (p = 0.010), a high educational level (p = 0.003), NOT having had a stroke (p = 0.025), and NOT having elevated blood lipids (p = 0.049). Model 5 shows the same associations with a strong additive effect of mental demands and education (p < 0.001) – indicating that having had a job with high mental demands and having a high educational level is associated with a better cognitive functioning in old age.

**Table 6:** Generalized linear model analysis of mental demands at work before retirement on cognitive functioning in old age (65+ years)

	**Model 4**			**Model 5**	
	**MMSE**			**MMSE**	
	** β**	** p**		** β**	** p**
Constant	3.284	0.000	Constant	3.261	0.000
Demands (high)	*0.036*	*0.010*	Demands & education (high & high)	*0.082*	*0.000*
Demands (medium)	*0.027*	*0.031*	Demands & education (high & middle)	*0.072*	*0.000*
Demands (low)	REF		Demands & education (middle & middle OR high & low)	*0.046*	*0.005*
			Demands & education (middle & low)	*0.052*	*0.002*
Age (70–79 years)	0.002	0.734	Demands & education (low & low)	REF	
Age (60–69 years)	REF				
Female	0.010	0.111	Age (70–79)	0.002	0.727
Male	REF		Age (60–69)	REF	
Self-regulation (high)	0.026	0.100	Female	0.011	0.075
Self-regulation (medium)	0.014	0.229	Male	REF	
Self-regulation (low)	REF		Self-regulation (high)	0.027	0.082
Education (high)	*0.023*	*0.003*	Self-regulation (medium)	0.013	0.248
Education (medium)	−0.002	0.767	Self-regulation (low)	REF	
Education (low)	REF		Stroke (yes)	*−0.041*	*0.021*
Stroke (yes)	*−0.040*	*0.025*	Stroke (no)	*REF*	
Stroke (no)	REF		Blood lipids (elevated)	0.011	0.060
Blood lipids (elevated)	*0.012*	*0.049*	Blood lipids (normal)	REF	
Blood lipids (normal)	REF		Liver disease (yes)	0.003	0.726
Liver disease (yes)	0.003	0.724	Liver disease (no)	REF	
Liver disease (no)	REF		Heart attack (yes)	−0.009	0.631
Heart attack (yes)	−0.014	0.478	Heart attack (no)	REF	
Heart attack (no)	REF		Blood pressure (high)	−0.002	0.769
Blood pressure (high)	−0.002	0.745	Blood pressure (normal)	REF	
Blood pressure (normal)	REF		Diabetes (yes)	0.000	0.962
Diabetes (yes)	−0.001	0.881	Diabetes (no)	REF	
Diabetes (no)	REF				

*Notes:* β - regression coefficient, *MMSE* mini-mental status examination, p - level of significance; *REF* reference category.

## Discussion

Our study aimed at examining the association between mental demands at work and cognitive functioning in the general population. Univariate as well as multivariate findings consistently demonstrated significant associations between higher mental demands and a better performance in cognitive tests. The results also indicate that the effects are independent of education. The data revealed no interaction effects but additive effects, meaning that high mental demands at work seem to be associated with cognitive functioning *additionally* to the level of education. Moreover, analyses of retired individuals confirmed a significant association between high mental demands at work and a better cognitive functioning in old age. Hence, it may be possible that jobs with high mental demands may constitute a beneficial factor for cognitive functioning in old age.

A possible explanation for the observed findings is that high mental demands at work might function like a training of cognitive abilities. A great number of studies indicated that cognitive abilities generally can be trained (e.g., [33,34]). Training effects, however, strongly depend on the level of demands: it has been demonstrated experimentally that only in high demand conditions participants can actually improve their performance [35]. Such experimental findings are also supported by findings of imaging studies showing that the training of highly demanding tasks obviously increases the activity of the fronto-parietal network in the brain and strengthens fronto-parietal as well as parietal-striatal connections [36,37]. As previous findings suggest a connection between the training of tasks with high mental demands and improved neural network efficiency, longitudinal studies are necessary in order to evaluate whether the association between high mental demands at work and a better cognitive functioning is subject to this particular effect as well.

Having a job with high mental demands might additionally contribute to a good cognitive reserve which protects cognitive functioning in old age. A good cognitive reserve is built up by the confrontation with mentally demanding conditions throughout the life-course, for example, obtaining a higher level of education [38]. Being confronted with high mental demands in the workplace for quite a long period of time may also constitute a life-course condition that builds up a cognitive reserve. Neuroimaging studies generally observed that with older age, the connectivity of the neural network in frontal brain regions gets disrupted [39]. Having a good cognitive reserve, however, seems to guard the brain against these age-related changes as neural imaging studies showed that older individuals with a higher cognitive reserve have a larger cortical thickness [40], a larger gray matter volume [41], and more efficiently functioning neural networks [42,43] than individuals of the same age but with a smaller cognitive reserve. A cognitive training that ameliorates the efficiency of the neural network – maybe in the form of high mental demands at work – might help building up a cognitive reserve and, hence, prolong the lifetime period during which we function well and stay symptom-free (compression of morbidity [44]). Further studies will have to validate this hypothesis.

Cognitive functioning, especially in old age, is also influenced by other factors. One important factor influencing old-age cognitive functioning is cardiovascular health. In our analysis, we have adjusted for cardiovascular factors. The association between high mental demands at work and a better cognitive functioning, however, was not modified by cardiovascular risk factors like heart attack, high blood pressure, or stroke. The prevalence of cardiovascular risk factors was similar across the different levels of mental demands at work, suggesting that the association between mental demands at work and cognitive functioning is independent of cardiovascular risk factors. Notwithstanding, further studies should investigate potentially mediating effects in more detail.

Our study is not without limitations. First, the study provides only cross-sectional findings. Longitudinal data are necessary to confirm the assumed causal association between high mental demands at work and better cognitive functioning. Secondly, it is unclear in how far a reversed causality might influence the results. Individuals may self-select or be recruited for particular occupations based on their capacities which then could reflect their performance. Only a longitudinal study design can reveal more details. In the present study, we adjusted the analysis for intelligence as measured by the German Version of the Mill Hill Vocabulary Scale. However, the vocabulary scale is only a crude proxy for intelligence. Consideration should also be given to the fact that occupational mental demands were exclusively operationalized by objective classification measures (O*NET descriptor variables). These measures, however, might differ from subjectively perceived levels of demands, as the latter strongly depend on factors like personality [45] and individual stress coping capacities [46]. Including subjective measures as well might thus help differentiating the general beneficial effects of high mental demands from those situations when high mental demands lead to decrements in cognitive performance. More research is needed to clearly understand the synergistic effect of mental demands with other influencing factors on an individual’s cognitive functioning.

A general methodological challenge arising in population-based studies is the problem of multicollinearity. In the present study we attempted to avoid problems of multicollinearity by (i) categorizing dependent variables, (ii) using a very large sample size, and (iii) combining dependent variables into one variable. However, we cannot be sure that we have completely ruled out any confounding.

In sum, our findings suggest a significant association between high mental demands at work and better cognitive functioning. In this sense, higher levels of mental demands – as brought about by technological changes in the working environment – may also have beneficial effects for the society as they could increase cognitive capacity levels and might even delay cognitive decline in old age. With this in mind, the debate about changes and challenges in the world of work should give a strong consideration to such potentially beneficial effects as well.

## Appendix

O*NET descriptor variables included in the index “mental demands”

1.A.1 Cognitive Abilities

1.A.1.a Verbal Abilities

1.A.1.a.1 Oral Comprehension: The ability to listen to and understand information and ideas presented through spoken words and sentences.

1.A.1.a.2 Written Comprehension: The ability to read and understand information and ideas presented in writing.

1.A.1.a.3 Oral Expression: The ability to communicate information and ideas in speaking so others will understand.

1.A.1.a.4 Written Expression: The ability to communicate information and ideas in writing so others will understand.

1.A.1.b Idea Generation and Reasoning Abilities

1.A.1.b.1 Fluency of Ideas: The ability to come up with a number of ideas about a topic (the number of ideas is important, not their quality, correctness, or creativity).

1.A.1.b.2 Originality: The ability to come up with unusual or clever ideas about a given topic or situation, or to develop creative ways to solve a problem.

1.A.1.b.3 Problem Sensitivity: The ability to tell when something is wrong or is likely to go wrong. It does not involve solving the problem, only recognizing there is a problem.

1.A.1.b.4 Deductive Reasoning: The ability to apply general rules to specific problems to produce answers that make sense.

1.A.1.b.5 Inductive Reasoning: The ability to combine pieces of information to form general rules or conclusions (includes finding a relationship among seemingly unrelated events).

1.A.1.b.6 Information Ordering: The ability to arrange things or actions in a certain order or pattern according to a specific rule or set of rules (e.g., patterns of numbers, letters, words, pictures, mathematical operations).

1.A.1.b.7 Category Flexibility: The ability to generate or use different sets of rules for combining or grouping things in different ways.

1.A.1.c Quantitative Abilities

1.A.1.c.1 Mathematical Reasoning: The ability to choose the right mathematical methods or formulas to solve a problem.

1.A.1.c.2 Number Facility: The ability to add, subtract, multiply, or divide quickly and correctly.

1.A.1.d Memory

1.A.1.d.1 Memorization: The ability to remember information such as words, numbers, pictures, and procedures.

1.A.1.e Perceptual Abilities

1.A.1.e.1 Speed of Closure: The ability to quickly make sense of, combine, and organize information into meaningful patterns.

1.A.1.e.2 Flexibility of Closure: The ability to identify or detect a known pattern (a figure, object, word, or sound) that is hidden in other distracting material.

1.A.1.e.3 Perceptual Speed: The ability to quickly and accurately compare similarities and differences among sets of letters, numbers, objects, pictures, or patterns. The things to be compared may be presented at the same time or one after the other. This ability also includes comparing a presented object with a remembered object.

1.A.1.f Spatial Abilities

1.A.1.f.1 Spatial Orientation: The ability to know your location in relation to the environment or to know where other objects are in relation to you.

1.A.1.f.2 Visualization: The ability to imagine how something will look after it is moved around or when its parts are moved or rearranged.

1.A.1.g Attentiveness

1.A.1.g.1 Selective Attention: The ability to concentrate on a task over a period of time without being distracted.

1.A.1.g.2 Time Sharing: The ability to shift back and forth between two or more activities or sources of information (such as speech, sounds, touch, or other sources).

## Competing interests

The authors declare that they have no competing interests.

## Authors’ contributions

FST has been involved in the acquisition of data, analyzed and interpreted the data, and wrote the first draft. TL has been involved in the acquisition of data, supervised the study, analyzed and interpreted the data, and critically commented and revised the manuscript. MLu supervised the study, analyzed and interpreted the data, and critically commented and revised the manuscript. KA has been involved in the acquisition of data and critically commented and revised the manuscript. MLS has been involved in the acquisition of data and critically commented and revised the manuscript. CE has been involved in the acquisition of data and critically commented and revised the manuscript. MLö conceived and designed the study, and critically commented and revised the manuscript. JT conceived and designed the study, and critically commented and revised the manuscript. AV conceived and designed the study, and critically commented and revised the manuscript. SGRH conceived and designed the study, supervised the study, analyzed and interpreted the data, and critically commented and revised the manuscript. All authors read and approved the final version of the manuscript.

## References

[B1] Handel MJ Organisation for Economic Co-operation and Development Trends in job skill demands in OECD countries OECD Social, Employment and Migration Working Papers 2012 143 1–119

[B2] Johnson RW Trends in job demands among older workers, 1992–2002 Mon Labor Rev 2004 127 48 56

[B3] Bundesverband BKK Kein Stress mit dem Stress: Eine Handlungshilfe für Betriebs- und Personalräte 2013 Dortmund: Bundesanstalt für Arbeitsschutz und Arbeitsmedizin

[B4] Galy E Cariou M Melan C What is the relationship between mental workload factors and cognitive load types? Int J Psychophysiol 2012 83 269 275 10.1016/j.ijpsycho.2011.09.023 22008523

[B5] Gonzalez C Task workload and cognitive abilities in dynamic decision making Hum Factors 2005 47 92 101 10.1518/0018720053653767 15960089

[B6] Hancock PA Williams G Manning CM Miyake S Influence of task demand characteristics on workload and performance Int J Aviat Psychol 1995 5 63 86 10.1207/s15327108ijap0501_5 11541497

[B7] Conrad CD A critical review of chronic stress effects on spatial learning and memory Prog Neuropsychopharmacol Biol Psychiatry 2010 34 742 755 10.1016/j.pnpbp.2009.11.003 19903505

[B8] Lupien SJ Maheu F Tu M Fiocco A Schramek TE The effects of stress and stress hormones on human cognition: Implications for the field of brain and cognition Brain Cogn 2007 65 209 237 10.1016/j.bandc.2007.02.007 17466428

[B9] Bosma H van Boxtel MPJ Ponds RWHM Houx PJ Burdorf A Jolles J Mental work demands protect against cognitive impairment: MAAS prospective cohort study Exp Aging Res 2002 29 33 45 12735080 10.1080/03610730303710

[B10] Potter GG Plassman BL Helms MJ Foster SM Edwards NW Occupational characteristics and cognitive performance among elderly male twins Neurology 2006 67 1377 1382 10.1212/01.wnl.0000240061.51215.ed 17060563

[B11] Seidler A Nienhaus A Bernhardt T Kauppinen T Elo AL Frolich L Psychosocial work factors and dementia Occup Environ Med 2004 61 962 971 10.1136/oem.2003.012153 15550601 PMC1740682

[B12] Smyth KA Fritsch T Cook TB McClendon MJ Santillan CE Friedland RP Worker functions and traits associated with occupations and the development of AD Neurology 2004 63 498 503 10.1212/01.WNL.0000133007.87028.09 15304581

[B13] Bonaiuto S Rocca WA Lippi A Giannandrea E Mele M Cavarzeran F Amaducci L Education and occupation as risk-factors for dementia - a population-based case–control study Neuroepidemiology 1995 14 101 109 10.1159/000109785 7777124

[B14] Fratiglioni L Winblad B von Strauss E Prevention of Alzheimer's disease and dementia. Major findings from the Kungsholmen project Physiol Behav 2007 92 98 104 10.1016/j.physbeh.2007.05.059 17588621

[B15] Karp A Andel R Parker MG Wang HX Winblad B Fratiglioni L Mentally stimulating activities at work during midlife and dementia risk after age 75: follow-up study from the Kungsholmen project Am J Geriatr Psychiatr 2009 17 227 236 10.1097/JGP.0b013e318190b691 19454849

[B16] Kröger E Andel R Lindsay J Benounissa Z Verreault R Laurin D Is complexity of work associated with risk of dementia? The Canadian Study of Health And Aging Am J Epidemiol 2008 167 820 830 10.1093/aje/kwm382 18263600

[B17] Stern Y What is cognitive reserve? Theory and research application of the reserve concept J Int Neuropsychol Soc 2002 8 448 460 10.1017/S1355617702813248 11939702

[B18] Forstmeier S Maercker A Motivational reserve: lifetime motivational abilities contribute to cognitive and emotional health in old age Psychol Aging 2008 23 886 899 19140658 10.1037/a0013602

[B19] Forstmeier S Maercker A Maier W van den Bussche H Riedel-Heller S Kaduszkiewicz H Pentzek M Weyerer S Bickel H Tebarth F Luppa M Wollny A Wiese B Wagner M Motivational reserve: motivation-related occupational abilities and risk of mild cognitive impairment and alzheimer disease Psychol Aging 2012 27 353 363 21875213 10.1037/a0025117

[B20] Aebi C Monsch AU Berres M Brubacher D Staehelin HB Validation of the German CERAD-neuropsychological assessment battery Neurobiol Aging 2002 23 S27 S28 10.1016/S0197-4580(01)00310-4

[B21] Chandler MJ Lacritz LH Hynan LS Barnard HD Allen G Deschner M Weiner MF Cullum CM A total score for the CERAD neuropsychological battery Neurology 2005 65 102 106 10.1212/01.wnl.0000167607.63000.38 16009893

[B22] Satzger W Hampel H Padberg F Burger K Nolde T Ingrassia G Engel RR Practical application of the CERAD test battery in screening for neuropsychological dementia Nervenarzt 2001 72 196 203 10.1007/s001150050739 11268764

[B23] Sanchez-Cubillo I Perianez JA Adrover-Roig D Rodriguez-Sanchez JM Rios-Lago M Tirapu J Bercelo F Construct validity of the Trail Making Test: role of task-switching, working memory, inhibition/interference control, and visuomotor abilities J Int Neuropsychol Soc 2009 15 438 450 10.1017/S1355617709090626 19402930

[B24] Salthouse TA What cognitive abilities are involved in trail-making performance? Intelligence 2011 39 222 232 10.1016/j.intell.2011.03.001 21789028 PMC3141679

[B25] Etnier JL Chang YK The effect of physical activity on executive function: a brief commentary on definitions, measurement issues, and the current state of the literature J Sport Exerc Psychol 2009 31 469 483 19842543 10.1123/jsep.31.4.469

[B26] Arbuthnott K Frank J Trail making test, part B as a measure of executive control: validation using a set-switching paradigm J Clin Exp Neuropsychol 2000 22 518 528 10.1076/1380-3395(200008)22:4;1-0;FT518 10923061

[B27] Drane DL Yuspeh RL Huthwaite JS Klingler LK Demographic characteristics and normative observations for derived-trail making test indices Neuropsychiatry Neuropsychol Behav Neurol 2002 15 39 43 11877550

[B28] Kraan C Stolwyk RJ Testa R The abilities associated with verbal fluency performance in a young, healthy population are multifactorial and differ across fluency variants Appl Neuropsychol: Adult 2013 20 159 168 10.1080/09084282.2012.670157 23383872

[B29] Henry JD Crawford JR Phillips LH Verbal fluency performance in dementia of the AlzheimerGÇÖs type: a meta-analysis Neuropsychologia 2004 42 1212 1222 10.1016/j.neuropsychologia.2004.02.001 15178173

[B30] Tombaugh TN Kozak J Rees L Normative data stratified by age and education for two measures of verbal fluency: FAS and animal naming Arch Clin Neuropsychol 1999 14 167 177 14590600

[B31] Parks RW Loewenstein DA Dodrill KL Barker WW Yoshii F Chang JY Emran A Apicella A Sheramata WA Duara R Cerebral metabolic effects of a verbal fluency test - a pet scan study J Clin Exp Neuropsychol 1988 10 565 575 10.1080/01688638808402795 3265709

[B32] Schmidt KH Metzler P Wortschatztest (WST) 1992 Göttingen: Hogrefe-Verlag

[B33] Tranter LJ Koutstaal W Age and flexible thinking: An experimental demonstration of the beneficial effects of increased cognitively stimulating activity on fluid intelligence in healthy older adults Aging Neuropsychol Cognit 2008 15 184 207 10.1080/13825580701322163 17851980

[B34] Jaeggi SM Buschkuehl M Jonides J Perrig WJ Improving fluid intelligence with training on working memory Proc Natl Acad Sci U S A 2008 105 6829 6833 10.1073/pnas.0801268105 18443283 PMC2383929

[B35] Fairclough SH Venables L Tattersall A The influence of task demand and learning on the psychophysiological response Int J Psychophysiol 2005 56 171 184 10.1016/j.ijpsycho.2004.11.003 15804451

[B36] Jolles DD Grol MJ Van Buchem MA Rombouts SARB Crone EA Practice effects in the brain: changes in cerebral activation after working memory practice depend on task demands Neuroimage 2010 52 658 668 10.1016/j.neuroimage.2010.04.028 20399274

[B37] Mackey AP Singley ATM Bunge SA Intensive reasoning training alters patterns of brain connectivity at rest J Neurosci 2013 33 4796 4803 10.1523/JNEUROSCI.4141-12.2013 23486950 PMC3657728

[B38] Valenzuela MJ Sachdev P Brain reserve and dementia: a systematic review Psychol Med 2006 36 441 454 16207391 10.1017/S0033291705006264

[B39] Chou YH Chen NK Madden DJ Functional brain connectivity and cognition: effects of adult age and task demands Neurobiol Aging 2013 34 1925 1934 10.1016/j.neurobiolaging.2013.02.012 23523269 PMC3674832

[B40] Liu YW Julkunen V Paajanen T Westman E Wahlund LO Aitken A Sobow T Mecocci P Tsolaki M Vellas B Muehlboeck S Spenger C Lovestone S Simmons A Soininen H Education increases reserve against Alzheimer's disease-evidence from structural MRI analysis Neuroradiology 2012 54 929 938 10.1007/s00234-012-1005-0 22246242 PMC3435513

[B41] Bartres-Faz D Sole-Padulles C Junque C Rami L Bosch B Bargallo N Falcon C Sanchez-Valle R Molinuevo JL Interactions of cognitive reserve with regional brain anatomy and brain function during a working memory task in healthy elders Biol Psychol 2009 80 256 259 10.1016/j.biopsycho.2008.10.005 19022337

[B42] Bastin C Yakushev I Bahri MA Fellgiebel A Eustache F Landeau B Scheurich A Feyers D Collette F Chetelat G Salmon E Cognitive reserve impacts on inter-individual variability in resting-state cerebral metabolism in normal aging Neuroimage 2012 63 713 722 10.1016/j.neuroimage.2012.06.074 22796505

[B43] Ansado J Monchi O Ennabil N Deslauriers J Jubault T Faure S Joanette Y Coping with task demand in aging using neural compensation and neural reserve triggers primarily intra-hemispheric-based neurofunctional reorganization Neurosci Res 2013 75 295 304 10.1016/j.neures.2013.01.012 23453977

[B44] Fries JF Bruce B Chakravarty E Compression of morbidity 1980-2011: a focused review of paradigms and progress J Aging Res 2011 261702 1 10 10.4061/2011/261702 PMC3163136 21876805

[B45] Conard MA Matthews RA Modeling the stress process: personality eclipses dysfunctional cognitions and workload in predicting stress Personal Individ Differ 2008 44 171 181 10.1016/j.paid.2007.07.023

[B46] Epstein S Katz L Coping ability, stress, productive load, and symptoms J Pers Soc Psychol 1992 62 813 825 1593421 10.1037//0022-3514.62.5.813

